# Impulse control disorders and their relationship with psychopathology in patients treated with cabergoline for hyperprolactinaemia

**DOI:** 10.1007/s11102-026-01693-7

**Published:** 2026-05-18

**Authors:** Luigi Zerbinati, Giulia Cristilli, Beatrice Valier, Francesca Braccia, David Okai, Tommaso Toffanin, Martina Verrienti, Antonella Giampietro, Luigi Grassi, Maria Chiara Zatelli, Maria Rosaria Ambrosio

**Affiliations:** 1 Department of Neuroscience and Rehabilitation, Institute of Psychiatry, University of Ferrara, Via Fossato Di Mortara 64A, Ferrara, Italy; 2 Department of Mental Health, University Hospital Psychiatric Unit, Local Health Trust, Ferrara, Italy; 3 Section of Endocrinology, Geriatrics and Internal Medicine, Department of Medical Sciences, University of Ferrara, Ferrara, Italy; 4 Department of Neuropsychiatry, South London and Maudsley NHS Foundation Trust, London, UK; 5 Unit of Endocrinology and Metabolic Diseases, Department of Specialty Medicine, Azienda Ospedaliero Universitaria S. Anna, Ferrara, Italy; 6 School of Medicine and Surgery, Università Cattolica del Sacro Cuore, Rome, Italy; 7 Department of Medicine, Endocrinology and Diabetology, Fondazione Policlinico Universitario A. Gemelli IRCCS, Largo A.Gemelli, Rome, Italy; 8 Present Address: ASL Città di Torino – S.C. Endocrinologia e Malattie del Metabolismo, Torino, Italy; 9 Present Address: Azienda USL di Imola – UOC Servizio Psichiatrico di Diagnosi e Cura, Imola, Italy; 10 Present Address: ASST Papa Giovanni XXIII, Bergamo, Italy

**Keywords:** Cabergoline, Dopamine agonist, Impulse control disorder, Impulse control behavior, Prolactine

## Abstract

**Purpose:**

Dopamine agonists (DAs), such as cabergoline (CAB), are first-line treatments for hyperprolactinaemia but have been associated with impulse control disorders (ICD). This observational study aimed to investigate the prevalence, severity, and clinical characteristics of ICD in CAB-treated patients compared with controls, using a combined clinician-administered and multimethod self-report assessment.

**Methods:**

A total of 126 participants were included: 63 patients with prolactin-secreting pituitary adenomas receiving cabergoline (CAB) and 63 controls (CG). ICD were evaluated using the Parkinson’s Impulse Control Scale (PICS) as the reference diagnostic measure, alongside self-report questionnaires assessing ICD-related behaviors. Psychiatric comorbidity was assessed using the Mini International Neuropsychiatric Interview (MINI), and general psychopathology using the Brief Symptom Inventory-53 (BSI-53).

**Results:**

ICD were identified in 15.9% of CAB-treated patients and 6.3% of controls, indicating a more than twofold higher prevalence and greater severity in the CAB group, although differences were not statistically significant. When restricted to CAB-related cases, prevalence decreased to 12.7%. The Questionnaire for Impulsive-Compulsive Disorders (QUIP) showed moderate sensitivity (71.4%) but low positive predictive value (41.7%). Within the CAB group, ICD-positive patients had shorter treatment duration and lower weekly doses. No significant differences emerged in MINI or BSI-53 results, although psychiatric comorbidity was present in ~ 50% of ICD cases.

**Conclusion:**

CAB therapy was associated with a modest but clinically meaningful increase in ICD. Screening tools may support early identification, but positive findings require confirmation through comprehensive clinical assessment.

**Supplementary information:**

The online version contains supplementary material available at 10.1007/s11102-026-01693-7.

## Introduction

Dopamine agonists (DAs) are the first-line treatment for prolactin-secreting pituitary adenomas, with cabergoline being the most commonly prescribed agent because of its high efficacy and favorable tolerability profile [1]. Increasing attention has focused on impulse control disorders (ICD) as a potential adverse effect of dopaminergic therapy, initially described in neurological settings [2] and more recently also in patients treated with dopamine agonists for prolactin-secreting pituitary adenomas [3].

ICDs refer to maladaptive, reward-driven behaviors, such as pathological gambling, compulsive shopping, binge eating, hypersexuality, or repetitive behaviors, that may lead to significant personal or social harm. While the association between dopamine agonists and ICD is well established in Parkinson’s disease, their occurrence during cabergoline treatment in endocrine disorders remains less clearly defined. Reported prevalence estimates in patients with hyperprolactinaemia vary widely, likely reflecting substantial heterogeneity in study design and assessment methods [4]. Most available studies rely predominantly on self-report screening questionnaires, whereas clinician-administered psychiatric assessments are uncommon, potentially contributing to both under-recognition in clinical practice and overestimation due to false-positive screening results [5–7].

More recently, studies incorporating structured or semi-structured interviews have reported more conservative prevalence estimates. In a cross-sectional study using a clinician-administered interview, ICD were identified in 12.7% of patients with dopamine agonist–treated secreting pituitary adenomas [8]. However, that study excluded individuals with current or past psychiatric disorders, a limitation of particular relevance given the frequent association between ICD and underlying psychopathology [9].

Taken together, these findings highlight the need for studies combining behavioral assessment with comprehensive psychiatric evaluation to better define the prevalence and clinical relevance of ICD during cabergoline therapy. Therefore, the present study aimed to evaluate ICD in patients with hyperprolactinaemia receiving cabergoline using a multimethod approach integrating self-report questionnaires with clinician-administered semi-structured interviews.

## Methods

### Participants

This multicentric observational study was conducted at three Italian referral centers for pituitary and psychiatric disorders: the Pituitary Disorders Clinic, A.O.U. S. Anna, Ferrara; the Department of Mental Health, University Hospital Psychiatric Unit, Ferrara; and the Hypothalamic–Pituitary Disorders Unit, Fondazione Policlinico Universitario A. Gemelli, Rome. The study was approved by the local Research Ethics Committees and conducted between May 2022 and September 2024.

The study population included adult patients (≥ 18 years) with pituitary adenomas associated with hyperprolactinaemia receiving cabergoline therapy. The control group consisted of patients with non-functioning pituitary adenomas without hyperprolactinaemia and healthy volunteers. Exclusion criteria were age < 18 years, current treatment with antipsychotics or mood stabilizers, hyperprolactinaemia due to non-pituitary causes, severe hepatic or renal disease, pregnancy or breastfeeding, previous cranial radiotherapy, intellectual disability or language barriers, inability or refusal to provide informed consent, and psychiatric disorders requiring active medical treatment.

Eligible participants were recruited during routine outpatient visits and provided written informed consent. All patients with pituitary adenomas underwent a standardized clinical evaluation performed by trained physicians. Collected data included demographic characteristics, lifestyle factors, tumour features, anthropometric measures, reproductive status in women, sexual dysfunction in men, serum prolactin levels, type and duration of DA therapy, current and cumulative cabergoline dose, and personal, family, and pharmacological history. Additional information was retrieved from electronic medical records.

For healthy volunteers, comparable demographic, clinical, and lifestyle information was collected through medical examination. After consent, all participants underwent a semi-structured psychiatric interview followed by completion of self-report questionnaires. The overall assessment required approximately 90 min.

### Measures

In this study, we consider the full spectrum of impulse control behaviors (ICB), encompassing both clinically significant manifestations and subthreshold symptoms. Although the literature sometimes differentiates impulse control disorders (ICD, defined as behaviors that substantially impair social or occupational functioning) from milder forms of ICB along a dimensional continuum, for clarity and consistency we use ICD to refer to the entire spectrum. Our analyses employ both a categorical and a dimensional approach, enabling us to assess not only the presence of clinically defined ICD but also variations in symptom severity across individuals.

#### Semi structured interviews

To assess the presence, severity, and functional impact of impulse control disorders, we administered the Parkinson’s Impulse Control Scale (PICS), a clinician-rated semi-structured interview [10]. The scale assesses multiple ICD domains, including compulsive medication use (dopamine dysregulation syndrome), gambling, shopping, eating, hypersexuality, and repetitive behaviors (punding and hobbyism). The interview includes an initial screening for the presence of ICD behaviors, as well as a separate item exploring their onset or worsening following DA exposure, when applicable. If the behavior is considered present, structured questions are used to rate both behavioral intensity, based on frequency and magnitude, and impact on personal and social functioning; these dimensions are combined to generate a global severity index. With patient consent, cases positive at interview in the treatment group underwent a confirmatory telephone assessment by an independent researcher to further evaluate the relationship with DA therapy.

The choice of this instrument was motivated by three main features: (1) the ability to provide a standardized measure of ICD severity; (2) its sensitivity to clinical changes over time, including DA tapering; and (3) the explicit evaluation of the relationship between ICD and DA use. As no validated Italian version of the instrument was available, a forward–backward translation was performed with the support of a native speaker. The procedure was carried out in collaboration with the original author of the scale (D.O.), who also provided online training in its administration.

Secondly, to evaluate the presence of current and past psychiatric disorders, we employed the Mini International Neuropsychiatric Interview (MINI 6.0) [11, 12], a structured diagnostic tool extensively validated for both DSM and ICD-10 classifications.

#### Self report measures

The Questionnaire for Impulsive-Compulsive Disorders in Parkinson’s Disease (QUIP) was administered as a self-report measure to screen for the presence of ICD. The QUIP is a yes/no screening tool organized into three sections: Sect. 1 explores four ICD (gambling, sexual, buying, and eating behaviors); Sect. 2 addresses other compulsive behaviors, including punding, hobbyism, and walkabout; and Sect. 3 evaluates compulsive medication use [13].

Hypersexuality was assessed using the Hypersexual Behavior Inventory (HBI), a validated instrument comprising three dimensions (control, coping, and consequences), rated on a four-point Likert scale from 1 (never) to 4 (very often) [14].

Pathological gambling was evaluated with the Gambling Urge Questionnaire (GUS), a six-item scale assessing gambling inclination, with responses scored from 0 (“strongly disagree”) to 7 (“strongly agree”) [15].

Compulsive eating was assessed using the Bulimia subscale of the Eating Disorder Inventory (EDI), Italian version; items are rated from never to always, with higher scores indicating greater severity. Item 53 (“I have thought about vomiting in order to lose weight”) was excluded [16].

Compulsive buying was evaluated using the Compulsive Buying Scale (CBS), a seven-item self-report measure assessing purchasing-related thoughts, emotions, and behaviors; a final score ≤ 1.34 indicates compulsive buying [17].

Lastly, general psychopathology was assessed using the Brief Symptom Inventory-53 (BSI-53), which evaluates symptoms experienced during the previous seven days across nine dimensions: hostility, depression, somatization, obsessive–compulsive symptoms, interpersonal sensitivity, anxiety, phobic anxiety, paranoid ideation, and psychoticism [18].

### Statistical analysis

An a priori power analysis was conducted using G*Power software (version 3.1), assuming an effect size (w) of 0.25, α = 0.05, and power (1 − β) = 0.80. The estimated sample size was 126 participants, equally divided between the cabergoline-treated (CAB) and control groups (CG) and matched for sex and age, based on the primary outcome of ICD presence.

Data were analyzed using SPSS (version 28). Statistical significance was set at *p* < 0.05 (two-tailed). Continuous variables are reported as means ± standard deviations, and categorical variables as counts and percentages. Between-group comparisons were performed using Student’s t-test or the Chi-square test, as appropriate. Bivariate analyses were conducted to explore associations, and graphical representations were generated using the ggplot package in R.

## Results

### Sample characteristics

Table 1 summarizes the sociodemographic and clinical characteristics of the study population. The final sample included 126 participants, equally divided between patients undergoing cabergoline therapy (CAB, *n* = 63) and a control group (CG, *n* = 63). Groups were matched for sex and age: in the CAB group 46.0% were male and 54.0% female, while in the CG 47.6% were male and 52.4% female, with a mean age of 48.0 years versus 48.7 years, respectively. Most participants in both groups were married or cohabiting (CAB: 71.4%; CG: 55.6%), had completed high school (CAB: 50.8%; CG: 46.0%), and were employed (CAB: 76.3%; CG: 66.7%).

**Table 1:** Socio-demographic and clinical characteristics of the sample

	CAB (*n*=63)	CG (*n*=63)	Total (*n*=126)
Age (mean ± DS)	48.7±14.2	48 ± 15.7	48.3 ± 14.9
Sex			
Male *n(%)*	29 (46%)	30 (47.6%)	59 (46.8%)
Female *n(%)*	34 (54%)	33 (52.4%)	67 (53.2%)
Marital Status			
Unmarried *n(%)*	13 (20.6%)	22 (34.9%)	35 (27.8%)
Married/living with a partner *n(%)*	45 (71.4%)	35 (55.6%)	80 (63.5%)
Divorced/widowed *n(%)*	5 (7.9%)	6 (9.5%)	11 (8.7%)
Education			
Primary school *n(%)*	2 (3.2%)	1 (1.6%)	3 (2.4%)
Middle school *n(%)*	9 (14.3%)	13 (20.6%)	22 (17.5%)
High school *n(%)*	32 (50.8%)	29 (46%)	61 (48.4%)
Bachelor’s degree *n(%)*	17 (27%)	13 (20.6%)	30 (23.8%)
Postgraduate/Master/PhD *n(%)*	3 (4.8%)	7 (11.1%)	10 (7.9%)
Working Status			
Student *n(%)*	1 (1.6%)	6 (9.5%)	7 (5.6%)
Housewife *n(%)*	0 (0%)	1 (1.6%)	1 (0.8%)
Retired *n(%)*	10 (15.9%)	10 (15.9%)	20 (15.9%)
Unemployed *n(%)*	4 (6.3%)	4 (6.3%)	8 (6.3%)
Employee *n(%)*	48 (76.3%)	42 (66.7%)	90 (71.4%)
BMI (mean ± DS)	27 ± 5	24.9 ± 4	25.9 ± 4.6
Clinical Characteristics of the sample (only Cabergoline Group)			
Age at onset (mean ± DS)	39.1 ± 15		
BMI at onset	26.8 ± 5.3		
Years of follow up (median + IQR)	9 (4-13.5)		
Years of therapy (median + IQR)	8 (3.25-12)		
PRL (ng/ml) (median + IQR)	9 (4.5–17.9)		
Weekly dose of cabergoline (mg) (median + IQR)	0.75 (0.5-1)		
Cumulative dose of cabergoline (mg) (median + IQR)	270.72 (102.4-499.9)		
Therapy with testosterone/estrogen-progestin *n (%)*	19 (30.2%)		

*Abbreviations: CAB *cabergoline*, CG* control group*, BMI *body mass index,* PRL* prolactine

Among CAB patients, prolactin levels at enrolment were within the normal range in almost all cases (median value 9 ng/ml). The median cabergoline dosage was relatively low (0.75 mg/week, IQR 0.5-1), with a cumulative median dose of 270.72 mg (IQR 102.4-499.9). Patients had been receiving therapy for a median duration of 8 years (IQR 3.25-12).

### Presence of ICD across the groups

In the overall sample, 24 participants screened positive for at least one ICD using the QUIP (QUIP+), of whom 14 were in the CG and 10 in the CAB group; this difference was not statistically significant (χ² = 0.82, df = 1, *p* = 0.36). By contrast, 14 participants were identified as positive with the PICS (PICS+), which was considered the gold standard. Among the 24 QUIP+ cases, 10 (41.7%) were also PICS + and 14 (58.3%) were PICS− (false positives). Conversely, among the 14 PICS+ participants, 10 (71.4%) were also QUIP+, whereas 4 (28.6%) were QUIP− (false negatives). Thus, when compared with the PICS, the QUIP showed a sensitivity of 71.4%, a specificity of 87.5%, a positive predictive value of 41.7%, and a negative predictive value of 96.1%. Among the 14 PICS+ patients, 10 were in the CAB group (10/63, 15.9%) and 4 in the CG (4/63, 6.3%). The difference approached but did not reach statistical significance (χ² = 2.89, df = 1, *p* = 0.08), indicating a trend toward a higher prevalence of ICD in the CAB group.

Table 2 reports the mean values of Intensity, Impact, and the combined Intensity × Impact scores for all ICD according to the PICS across the two groups. On the whole, the CAB group showed higher values in all dimensions, although none of these differences reached statistical significance (all *p* > 0.05).

**Table 2:** Results of QUIP and PICS questionnaires

	CAB	CG	Statistics
QUIP			
QUIP-Gambling	3 (3.2%)	1 (1.6%)	χ²=0.34; df = 1; *p* > 0.05
QUIP-Hypersexuality	3 (4.8%)	0 (0%)	χ²=3.07; df = 1; *p* > 0.05
QUIP-Buying	5 (6.3%)	2 (3.2%)	χ²=0.7; df = 1; *p* > 0.05
QUIP-Eating	4 (6.3%)	3 (4.8%)	χ²=0.15; df = 1; *p* > 0.05
QUIP-Hobbyism	2 (3.2%)	5 (7.9%)	χ²=1.36; df = 1; *p* > 0.05
QUIP-Punding	0 (0%)	4 (6.3%)	χ²=4.13; df = 1; *p* > 0.05
QUIP-Walkabout	0 (0%)	1 (1.6%)	χ²=1; df = 1; *p* > 0.05
QUIP-Medication	0 (0%)	0 (0%)	-
PICS			
PICS-Eating			
-Intensity	0.13 ± 0.52	0.03 ± 0.25	t=-1.3; df = 89.3; *p* > 0.05
-Impact	0.09 ± 0.39	0.01 ± 0.12	t= -1.5; df = 74.8; *p* > 0.05
-Intensity X Impact	0.19 ± 0.93	0.03 ± 0.25	t=-1.31; df = 71; *p* > 0.05
PICS-Gambling			
-Intensity	0.11 ± 0.54	0	t=-1.63; df = 62; *p* > 0.05
-Impact	0.06 ± 0.30	0	t=-1.65; df = 62; *p* > 0.05
-Intensity X Impact	0.16 ± 1.02	0	t=-1.24; df = 62; *p* > 0.05
PICS-Hypersexuality			
-Intensity	0.95 ± 0.39	0.48 ± 0.21	t=-0.85; df = 124; *p* > 0.05
-Impact	0.11 ± 0.48	0.03 ± 0.18	t=-1.23; df = 124; *p* > 0.05
-Intensity X Impact	0.19 ± 0.91	0.03 ± 0.18	t=-1.35; df = 124; *p* > 0.05
PICS-Buying			
-Intensity	0.05 ± 0.28	0	t=-1.35; df = 62; *p* > 0.05
-Impact	0.03 ± 0.18	0	t=-1.43; df = 62; *p* > 0.05
-Intensity X Impact	0.05 ± 0.28	0	t=-1.35; df = 62; *p* > 0.05
PICS- DDS/*off* period dysphoria			
-Intensity	0	0	
-Impact	0	0	
-Intensity X Impact	0	0	
PICS- Punding			
-Intensity	0	0.05 ± 0.28	t = 1.35; df = 62; *p* > 0.05
-Impact	0	0.05 ± 0.28	t = 1.35; df = 62; *p* > 0.05
-Intensity X Impact	0	0.08 ± 0.52	t = 1.22; df = 62; *p* > 0.05
PICS- Hobbyism			
-Intensity	0.09 ± 0.53	0.01 ± 0.12	t=-1.16; df = 69; *p* > 0.05
-Impact	0.05 ± 0.28	0	t=-1.35; df = 62; *p* > 0.05
-Intensity X Impact	0.14 ± 0.83	0	t=-1.35; df = 62; *p* > 0.05

*Abbreviations: CAB* cabergoline, *CG* control group, *QUIP* Questionnaire for Impulsive-Compulsive Disorders in Parkinson’s Disease, *PICS* Parkinson’s Impulse Control Scale

**Fig. 1:**
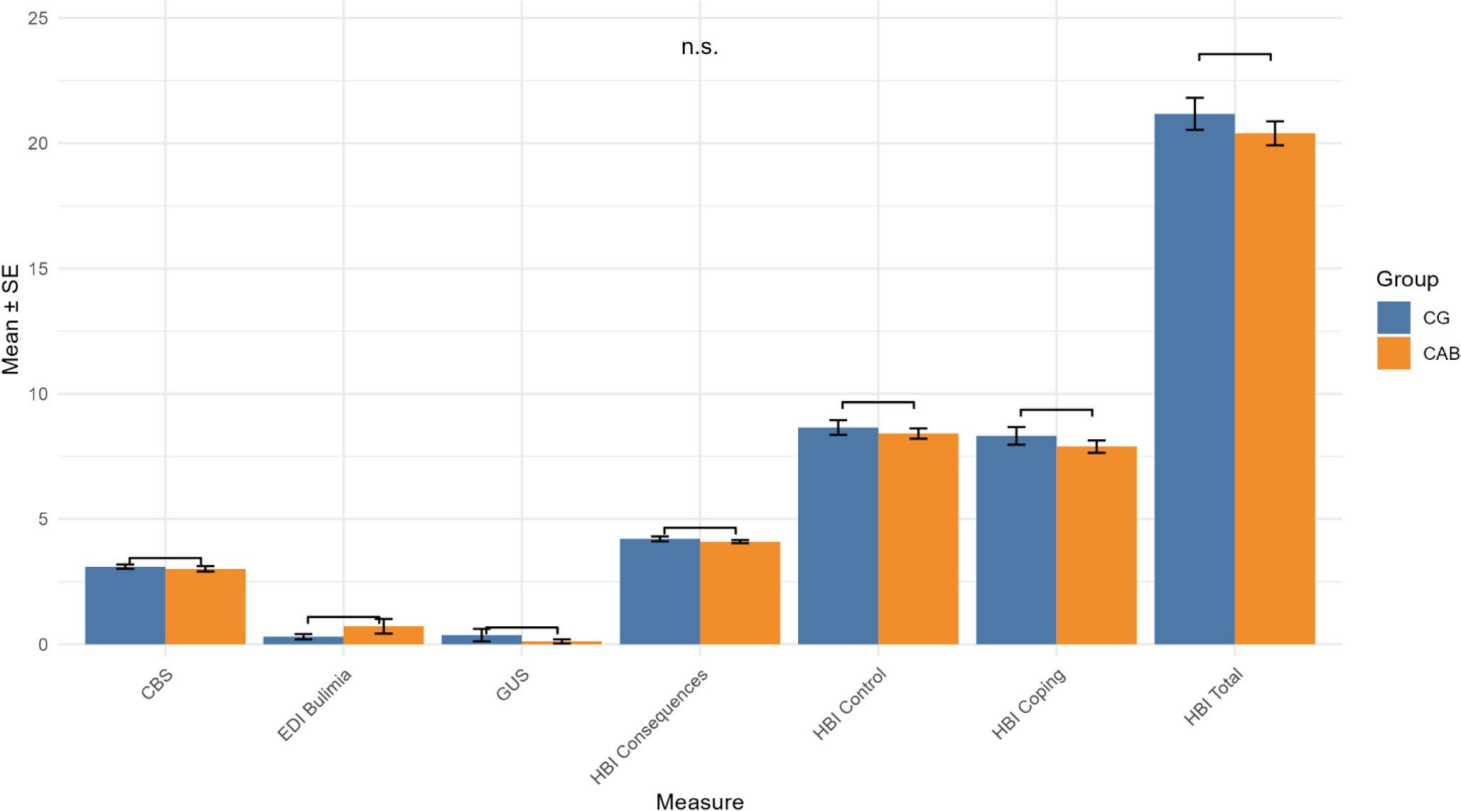
ICD self report scores by group. Abbreviations: CAB: cabergoline; CG: control group; CBS: Compulsive Buying Scale; EDI: Bulimia subscale of the Eating Disorder Inventory; GUS: Gambling Urge Questionnaire; HBI: Hypersexual Behavior Inventory

Table S1 and Fig. 1 present the mean scores of the self-report questionnaires assessing ICD dimensions. No significant differences were observed between the two groups.

Specifically within the CAB group, 10 patients were PICS+ (10/63, 15.87%). Of these, 8 clearly confirmed the onset or worsening of an ICD based on (1) targeted questions during the structured interview and (2) a subsequent telephone interview conducted by an independent, blinded researcher. In 2 of the 10 cases, a definitive link with cabergoline could not be established due to pre-existing psychopathology (see next section) or possible recall bias. Whenever feasible and with patient authorization, additional information was obtained by interviewing family members or partners. Thus, 8 out of 10 CAB/ICD+ patients (8/63, 12.7%) were judged to have clearly experienced a new onset or worsening of ICD (Table 3). Notably, among the 4 patients with hypersexuality, 2 were also receiving testosterone undecanoate depot (TUD); in one of these cases the contribution of CAB could not be clearly determined, whereas in the remaining 3 hypersexuality was consistently reported as a side effect since the initiation of therapy. In addition, one patient described an increase in compulsive buying following cabergoline treatment, two reported clear episodes of binge eating, one of whom was also at the onset of menopause, and two others indicated a worsening of gambling behavior. In cases where a temporal relationship with the drug was clearly established, patients generally reported an early onset of ICD symptoms following initiation of cabergoline therapy, typically within the first months. However, precise time-to-onset data could not be reliably determined, as this information was based on retrospective recall within a cross-sectional assessment.

**Table 3:** Individuals with diagnosed ICD

Group	Sex	Age	Weekly dose of cabergoline (mg)	Cumulative dose of cabergoline (mg)	PICS	Past psychiatric history	Actual psychiatric disorders (MINI)	Psychiatric family history	Testosterone therapy	Relation with cabergoline/ management/ other
CAB	Male	45	0.5	142	Compulsive eating: Intensity 2; Impact 1; IxI 2	Eating disorders	Possible Binge Eating Disorder	Yes	No	Unclear Clinical monitoring (no psychiatric referral required)
CAB	Male	51	0.75	288.2	Hypersexuality: Intensity 2; Impact 2; IxI 4	No	No	No	Yes	Hypersexuality since on cabergoline reported; symptoms no longer reported at endocrinology follow-up visits Total T: 4.19 ng/mL
CAB	Male	57	0.5	309	Hypersexuality: Intensity 2; Impact 3; IxI 6 Hobbyism: Intensity 3; Impact 2; IxI 6	No	No	No	Yes	Unclear; symptoms no longer reported at endocrinology follow-up visits Total T: 2.06 ng/mL
CAB	Male	65	0.5	94	Hypersexuality: Intensity 1; Impact 1; IxI 1 Hobbyism: Intensity 3; Impact 1; IxI 3	No	Generalized anxiety disorder Panic disorder	Yes	No	Hypersexuality since on cabergoline reported; symptoms no longer reported at endocrinology follow-up visits Total T: 4.37 ng/ml
CAB	Male	87	1.5	459	Hypersexuality: Intensity 1; Impact 1; IxI 1	No	No	No	No	Hypersexuality since on cabergoline reported; symptoms no longer reported at endocrinology follow-up visits Total T: 4.94 ng/ml
CAB	Female	24	0.5		Bulimia: Intensity 3; Impact 2; IxI 6 Gambling: Intensity 1; Impact 1; IxI 1 Compulsive buying: Intensity 1; Impact 1; IxI 1	No	Persistent depressive disorder Panic disorder Suicide risk	No		Binge eating episodes since on cabergoline reported; marked reduction after drug discontinuation Psychiatric outpatient care
CAB	Female	25	0.5	33	Gambling: Intensity 4; Impact 2; IxI:8	Generalized anxiety disorder	Panic disorder	Yes		Gambling intensified on cabergoline; drug discontinued Lost to follow ups (living in another city)
CAB	Female	40	0.25	493	Gambling: Intensity 1; Impact 1; IxI 1	Eating disorders Panic disorder	No	No		Gambling intensified on cabergoline; dose reduced to every 10 days, then discontinued; symptoms improved
CAB	Female	41	0.5	772.47	Compulsive buying: Intensity 2; Impact 1; IxI 2	No	Generalized anxiety disorder	No		More frequent buyings since on cabergoline reported Lost to follow ups
CAB	Female	48	0.5	43.5	Compulsive eating: Intensity 2; Impact 2; IxI 4	No	No	No		Binge eating intensified since on cabergoline but at the same time as the onset of menopause; dose reduced (every 15 days) no follow-up data available on eating symptoms
		48.3 ± 18.6	0.6 ± 0.3	255 ± 194.5						
CG	Male	34			Hypersexuality: Intensity 1; Impact 1; IxI 1 Punding: Intensity 1; Impact 1; IxI 1	No	Generalized anxiety disorder	Yes	No	
CG	Male	53			Hypersexuality: Intensity 1; Impact 1; IxI 1	No	No	No	No	
CG	Female	24			Punding: Intensity 2; Impact 2; IxI 4	No	No	No		
CG	Female	25			Compulsive eating: Intensity 2; Impact 1; IxI 4	Eating disorders	Persistent depressive disorder Panic disorder	No		
		34 ± 13.4								

*Abbreviations:* *CAB* Cabergoline, *CG* Control Group, *PICS* Parkinson’s Impulse Control Scale, *MINI* Mini International Neuropsychiatric Interview, *T* testosterone

With reference still to the CAB group, PICS+ patients had been on therapy for a shorter duration compared with PICS− patients (5.96 ± 3.17 vs. 8.98 ± 6.58 years; *t* = 2.23, df = 26.81, *p* = 0.01) and were receiving a lower weekly CAB dose (0.6 ± 0.33 mg vs. 1.05 ± 0.94 mg; *t* = 2.67, df = 40.33, *p* = 0.01). Although these differences did not reach statistical significance (all *p* > 0.1), PICS+ patients showed lower prolactine levels (11.72 ± 6.77 vs. 16.96 ± 24.67 ng/ml) and had received a lower cumulative CAB dose (292.68 ± 247.48 vs. 445.46 ± 520.20 mg) compared with PICS− patients.

In the control group, two patients presented with punding, one of whom also exhibited hypersexuality, resulting in a total of two cases of hypersexuality. In addition, one patient reported compulsive eating, although she had a past history of eating disorders.

### Psychopathology and its relationship with ICD

In the CAB group, among the 10 PICS+ patients, 4 (40.0%) also met criteria for a psychiatric disorder on the MINI interview (MINI+). Conversely, of the 11 MINI+ patients (17.5%), 4 (36.4%) were also PICS+ (χ² = 4.19, df = 1, *p* = 0.04). One patient was PICS + for compulsive eating but also had a history of eating disorder and was considered to have a possible current eating disorder. It should be noted that the MINI screens only for anorexia nervosa and bulimia nervosa, but does not include an assessment of binge eating disorder. Of the 8 patients with a clear drug-related worsening of ICD, 4 (50.0%) were also MINI+, 2 (25.0%) had a positive past history of psychiatric disorder, and 2 (25.0%) reported a positive family history of psychiatric disorder.

In the CG, among the 4 PICS+ patients, 2 (50.0%) were also MINI+. Of the 8 MINI+ patients (12.7%), 2 (25.0%) were also PICS+ (χ² = 5.36, df = 1, *p* = 0.02). Notably, one patient who was PICS + for compulsive eating also reported a past psychiatric history of eating disorders.

Table 4 presents the proportion of patients meeting MINI diagnostic criteria in the two groups, as well as the mean scores of self-reported psychopathological measures (also see supplementari Figure S1). Overall, no significant differences were observed either in the distribution of diagnoses or in the average scores of psychopathological dimensions between the two groups (all *p* > 0.05).

**Table 4:** MINI interview and BSI scores by group

	CAB	CG	Statistics
MINI-Affective	3 (4.8%)	4 (6.3%)	χ²=0.15; df=1; p>0.05
MINI- Anxiety	9 (14.3%)	7 (11.1%)	χ²=0.28; df = 1; *p* > 0.05
MINI-Other	1 (1.6%)	0 (0%)	χ²=1; df = 1; *p* > 0.05
Suicide Risk (present)	3 (4.8%)	2 (3.2%)	χ²=0.2; df = 1; *p* > 0.05
BSI-Somatization	0.3 ± 0.35	0.4 ± 0.58	t = 1.12; df = 124; *p* > 0.05
BSI-Obsessivity	0.43 ± 0.48	0.58 ± 0.58	t = 1.53; df = 123; *p* > 0.05
BSI-Interpersonal Sensitivity	0.4 ± 0.52	0.36 ± 0.45	t=-0.41; df = 124; *p* > 0.05
BSI-Depression	0.38 ± 0.47	0.4 ± 0.53	t = 0.18; df = 124; *p* > 0.05
BSI-Anxiety	0.4 ± 0.42	0.44 ± 0.46	t = 0.5; df = 124; *p* > 0.05
BSI-Hostility	0.33 ± 0.46	0.31 ± 0.4	t=-0.25; df = 124; *p* > 0.05
BSI-Phobic Anxiety	0.12 ± 0.22	0.11 ± 0.25	t=-0.15; df = 124; *p* > 0.05
BSI-Paranoia	0.33 ± 0.40	0.37 ± 0.47	t = 0.61; df = 124; *p* > 0.05
BSI-Psychoticism	0.18 ± 0.32	0.2 ± 0.34	t = 0.21; df = 124; *p* > 0.05

*Abbreviations*: *CAB *cabergoline, *CG* control group, *MINI *Mini International Neuropsychiatric Interview, *BSI* Brief Symptom Inventory

## Discussion

This case–control study investigated the prevalence and clinical features of ICDs in patients with hyperprolactinaemia treated with cabergoline, using a rigorous approach that combined self-report questionnaires with semi-structured psychiatric interviews.

A first general finding of the study was that 15.9% of patients in the CAB group exhibited an ICD, compared with 6.3% in the control group. When cases in which the relationship with cabergoline was uncertain or the worsening could not be clearly attributed to the drug were conservatively excluded, the prevalence decreased to 12.7% in the CAB group versus 6.3% in controls. Although this difference did not reach statistical significance, it showed a clear trend toward a higher frequency of ICD in the CAB group, approximately twice that observed in the control group. Interestingly, this frequency of ICD in our CAB sample was identical to that reported in another cross-sectional study using a structured clinical interview [8], while the prevalence in the control group was comparable to that observed in the general population [19]. In addition, we found greater ICD severity among patients receiving cabergoline, though this difference again failed to reach significance, likely due to the limited sample size. Taken together, these findings suggest a modest increase in both the frequency and intensity of ICD among cabergoline-treated patients. By contrast, studies relying on self-report instruments have often yielded much higher prevalence estimates. For instance, De Sousa et al. found that 61.1% of patients receiving dopamine agonists and 42.4% of healthy controls screened positive for at least one ICD using the QUIP—figures that diverge substantially from real-world clinical observations [6]. The relatively lower prevalence detected in our study, therefore, likely reflects the use of a clinician-administered structured interview, which allowed symptoms to be directly linked to cabergoline exposure and to better represent real-world clinical experience than self-report surveys alone. Moreover, in our sample, 58.3% of participants who screened positive on the QUIP did not meet ICD diagnostic criteria on the clinician-administered PICS, resulting in a markedly low positive predictive value (41.7%) and indicating limited agreement between the two measures. This finding is consistent with previous reports showing only moderate concordance (κ = 0.408) between self-reported and clinician-rated assessments of impulsive–compulsive behaviors [20, 21]. Overall, self-report questionnaires such as the QUIP represent valuable screening tools with excellent negative predictive value; however, a positive result should always be followed by a comprehensive clinical evaluation due to the high rate of false positives [21]. Our findings therefore point toward a more conservative and clinically realistic estimate of ICD frequency, consistent with the low rates typically observed in endocrinological practice.

Within the CAB group, an interesting finding emerged regarding the weekly dose of cabergoline, which was lower in patients presenting with ICD. Although most studies have not demonstrated a consistent association between ICD occurrence and the weekly dosage of cabergoline [22], the literature remains inconclusive. Our observation aligns with reports suggesting that ICD may develop even at relatively low doses of dopamine agonists. For instance, Davie described a case of pathological gambling emerging after only one year of cabergoline treatment at 0.25 mg per week, highlighting that even minimal dopaminergic exposure can trigger behavioral disturbances in susceptible individuals [23]. Similarly, another study reported that patients diagnosed with ICD exhibited a lower cumulative dose of cabergoline compared to those without ICD; the authors proposed that this pattern might reflect an increased vulnerability among DA-naïve patients or those in the early stages of therapy [24]. This interpretation is consistent with our data showing a shorter treatment duration among ICD patients. However, although cumulative cabergoline doses were also lower in our ICD subgroup, this difference did not reach statistical significance, possibily because of limited sample size. Finally, Penchev et al., in a recent meta-analysis, found that weekly dosage appeared to modulate the risk of hypersexuality, with a trend toward higher risk at lower doses (≤ 1 mg/week compared with 1.1–2 mg/week) [25]. While this finding may appear counterintuitive, since hypersexuality and other ICD are sometimes reported to improve following dose reduction [26], it nevertheless supports the hypothesis of a non-linear dose–response relationship. In fact, the emergence of ICD in patients treated with cabergoline is likely influenced by multiple interacting factors, including the drug’s complex pharmacodynamic profile. Cabergoline acts as a full agonist at D₂ receptors and a partial agonist at D₃ receptors, while also exhibiting affinity for several serotonergic sites [27]. In particular, its partial agonism at 5-HT₁A receptors could be implicated in modulating reward-related and impulsive behaviors. Experimental evidence indicates that stimulation of 5-HT₁A receptors, especially at lower doses [28], can promote reward-driven and impulsive behaviors in animal models [29–32], whereas receptor blockade exerts the opposite effect. Moreover, human studies suggest that genetic variability in 5-HT₁A receptor expression may confer individual susceptibility to impulsivity and related behavioral traits [33]. Similarly, polymorphisms in the D₃ receptor gene have been proposed as potential vulnerability factors for the development of drug-induced ICD [34]. In addition, genetic variability in drug transport across the blood–brain barrier may further contribute to interindividual susceptibility, as cabergoline is a substrate of P-glycoprotein (P-gp), encoded by the ABCB1 gene, and polymorphisms in this transporter have been associated with differences in central drug exposure and the occurrence of common central side effects [35]. Taken together, these findings support the view that cabergoline-induced behavioral changes may stem from the combined effects of dopaminergic D₃ receptor activation—facilitating approach and reward-seeking behavior—and 5-HT₁A receptor stimulation, which may attenuate serotonergic inhibitory control over impulsive actions [36], as well as from interindividual differences in blood–brain barrier permeability related to P-glycoprotein (P-gp) activity, encoded by the ABCB1 gene, which may influence central drug exposure.

Hormonal status appears to play an important role in the development of impulse control disorders, likely through its interaction with the mesolimbic dopaminergic system. Testosterone has been implicated in the regulation of impulsive and risk-taking behaviors, particularly in men. Increases in testosterone may modulate the prefrontal cortex and mesolimbic regions involved in reward and motivation, potentially disrupting the balance between inhibitory control and reward sensitivity [37]. In our CAB group, all four patients who developed hypersexuality were male, and two were receiving testosterone undecanoate. These patients showed higher *Intensity*, *Impact*, and *Intensity × Impact* scores than hypersexual CAB patients not on testosterone, supporting the hypothesis of a synergistic interaction between DA therapy and testosterone normalization or supplementation—a phenomenon described as “dopa-testotoxicosis” [26]. Interestingly, patients who reported hypersexuality at the time of psychiatric assessment subsequently described a normalization of symptoms at later endocrinological follow-up visits, occurring several months after the initial evaluation and the confirmatory telephone interview. This finding may reflect either a true resolution of symptoms or potential underreporting at follow-up, possibly related to stigma, or to a reduced perception of the behavior as problematic, or even to its subjective experience as rewarding or desirable.

On the other hand, female sex hormones may also play a role in the emergence of impulse control disorders during DA therapy. We observed the *de novo* onset of compulsive eating in a woman entering menopause, which markedly worsened following the initiation of cabergoline treatment. Fluctuations in ovarian hormones are known to influence dopaminergic activity and its regulation of reward-related impulsivity through effects on basal and frontal cortical circuits [38] ; furthermore, findings from different studies suggest that menopause is associated with marked structural and functional differences in the frontal regions, which are critical for inhibitory control [39]. Consistent with these neurobiological mechanisms, several studies have reported the onset or worsening of binge eating symptoms during the perimenopausal and menopausal phases, suggesting that hormonal instability may increase susceptibility to reward-driven behaviors [40, 41]. In this context, cabergoline, by further enhancing dopaminergic tone, may exacerbate these vulnerabilities. Indeed, binge eating has been described among the potential adverse effects of cabergoline therapy [42, 43] .

Finally, we observed that, among the ten patients treated with cabergoline who met criteria for an ICD, five (50%) presented with a comorbid psychiatric disorder. One patient with compulsive eating had a prior history of eating disorder, although the MINI interview does not screen for binge eating disorder, which may have led to an underestimation of this comorbidity. A possible link between ICD and psychopathology may stem from shared neurobiological vulnerabilities, such as serotoninergic hypofunction [37, 44] or genetic factors including polymorphisms in the serotonin transporter gene (5-HTTLPR) [45]. Alternatively, the high rate of comorbidity could reflect the psychological consequences of ICD themselves, such as shame, social withdrawal, low self-esteem, or guilt [46], which may contribute to the emergence or worsening of mood and anxiety symptoms. This finding is supported by the fact that we observed about the same prevalence of psychiatric comorbidities in the control group (50%). Future studies integrating neurobiological and psychopathological assessments are warranted to better clarify the nature of this association.

This study has some limitations. First, the sample size was relatively small, limiting the generalizability of the findings. In addition, the prevalence of ICD may be underestimated, as the PICS does not assess certain behaviors such as skin picking, intermittent explosive disorder, kleptomania, pyromania, or trichotillomania. Future studies should integrate structured interviews with additional measures of impulsivity, including neuropsychological tasks, to provide a more comprehensive assessment. The inclusion of a control group of patients with prolactinomas undergoing primary surgical treatment without prior dopamine agonist exposure would also represent a valuable direction for future research.

Despite these limitations, the study is strengthened by its methodological rigor, combining clinician-administered psychiatric interviews with validated self-report measures. The use of structured interviews and collateral information allowed a more accurate attribution of ICD to cabergoline exposure and helped clarify discrepancies between assessment tools, addressing an important source of heterogeneity in the existing literature.

## Conclusions

In conclusion, patients treated with cabergoline showed a higher frequency and greater severity of impulse control disorders, although these differences did not reach statistical significance. The observed prevalence appears more clinically realistic than estimates based solely on self-report questionnaires and is consistent with studies employing standardized clinical interviews. While self-report measures may be useful for screening purposes, accurate identification of impulse control disorders requires comprehensive clinical assessment. In fact, the prevalence of ICD in patients receiving cabergoline is likely influenced by multiple interacting factors, including treatment dose, individual impulsivity traits, underlying psychopathology, and hormonal status.

## Supplementary material

Below is the link to the electronic supplementary material.

Supplementary File 1 (PDF 122 KB)

## Data Availability

The datasets generated and/or analysed during the current study are not publicly available due to privacy and ethical restrictions but are available from the corresponding author on reasonable request.
