# Supplementary material for: Impulse control disorders and their relationship with psychopathology in patients treated with cabergoline for hyperprolactinaemia
Source: Pituitary. 2026 May 18;29(3):87. doi: 10.1007/s11102-026-01693-7 (PMC13183727; doi:10.1007/s11102-026-01693-7)
Supplement: Supplementary file 1 — (PDF 122 KB) [file 11102_2026_1693_MOESM1_ESM.pdf]

Table S1. ICD self report scores according to study group

	Cabergoline	Control Group	Statistics
**CBS**	0.6±1.2	0.42±0.1	t=-0.87, df=123, p>0.05
**EDI-Bulimia**	0.71±2.32	0.3±0.83	t=-1.33, df=77.8, p≤0.05
**GUS**	0.11±0.65	0.36±1.97	t=0.96, df=75.8, p≤0.05
**HBI-Consequences**	4.1±0.46	4.2±0.79	t= 0.96, df=124, p>0.05
**HBI-Control**	8.41±1.62	8.6±2.32	t= 0.67, df=124, p>0.05
**HBI-Coping**	7.88±1.97	8.32±2.76	t= 1, df=124, p>0.05
**HBI-Total**	20.4±3.8	21.2±5.08	t= 0.97, df=124, p>0.05

Abbreviations: CBS: Compulsive Buying Scale; EDI: Bulimia subscale of the Eating Disorder Inventory; GUS: Gambling Urge Questionnaire; HBI: Hypersexual Behavior Inventory; CAB: Cabergoline; CG: Control Group.

Figure S1. BSI-53 Scores by study group

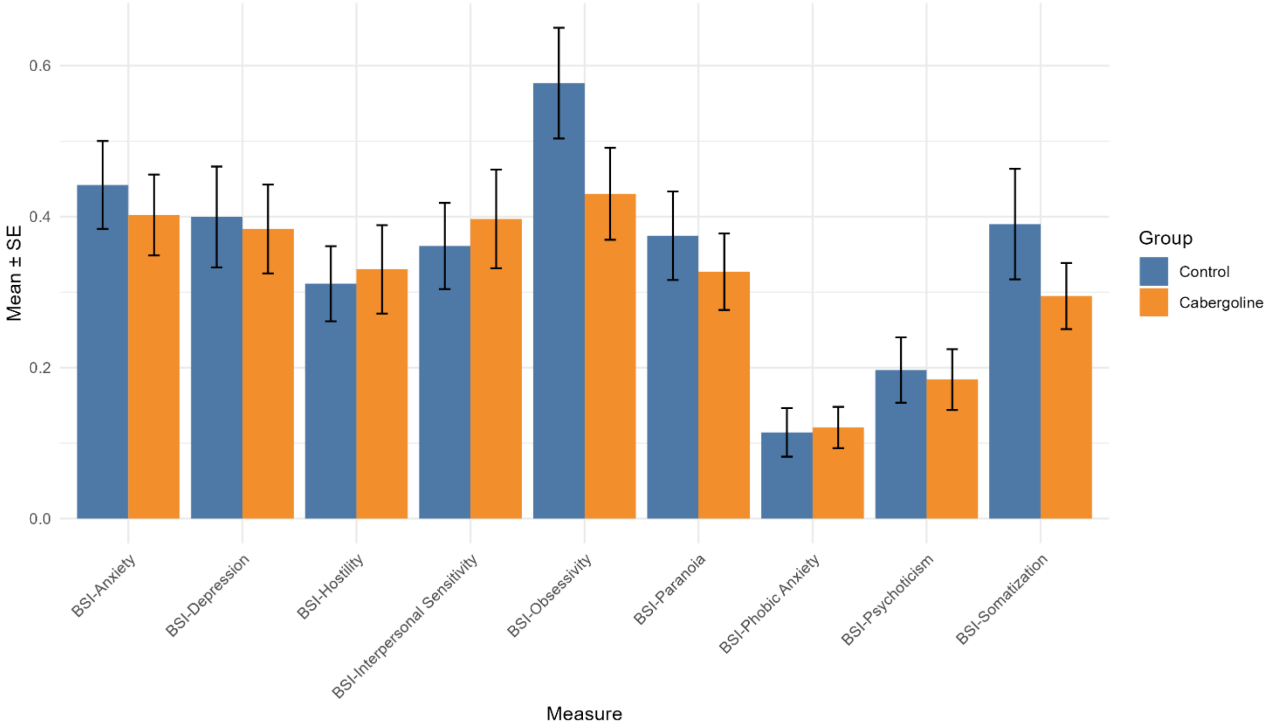

Abbreviations: CAB: cabergoline; CG: control group; BSI: Brief Symptom Inventory
